# Three Different Learning Curves Have an Independent Impact on Perioperative Outcomes After Robotic Partial Nephrectomy: A Comparative Analysis

**DOI:** 10.1245/s10434-020-08856-1

**Published:** 2020-07-24

**Authors:** Philip Zeuschner, Irmengard Meyer, Stefan Siemer, Michael Stoeckle, Gudrun Wagenpfeil, Stefan Wagenpfeil, Matthias Saar, Martin Janssen

**Affiliations:** 1 Department of Urology and Pediatric Urology, Saarland University, Homburg/Saar, Germany; 2 Department of Medical Biometry, Epidemiology and Medical Informatics, Saarland University, Homburg/Saar, Germany; 3 Present Address: Department of Urology and Pediatric Urology, University Hospital of Munster, Münster, Germany

## Abstract

**Background:**

Robot-assisted partial nephrectomy (RAPN) has become widely accepted, but its different underlying types of learning curves have not been comparatively analyzed to date. This study aimed to determine and compare the impact that the learning curve of the department, the console surgeon, and the bedside assistant as well as patient-related factors has on the perioperative outcomes of RAPN.

**Methods:**

The study retrospectively analyzed 500 consecutive transperitoneal RAPNs (2007–2018) performed in a tertiary referral center by 7 surgeons and 37 bedside assistants. Patient characteristics and surgical data were obtained. Experience (EXP) was defined as the current number of RAPNs performed by the department, the surgeon, and the assistant. As the primary outcome, the impact of EXP and patient-related factors on perioperative outcomes were analyzed and compared. As the secondary outcome, a cutoff between “experienced” and “inexperienced” was defined. Correlation and regression analysis, receiver operating characteristic curve analysis, Fisher’s exact test, and the Mann–Whitney *U* test were performed, with *p* values lower than 0.05 denoting significance.

**Results:**

The EXP of the department, the surgeon, and the assistant each has a major influence on perioperative outcome in RAPN irrespective of patient-related factors. Perioperative outcomes improve significantly with EXP greater than 100 for the department, EXP greater than 35 for the surgeon, and EXP greater than 15 for the assistant.

**Conclusions:**

The perioperative results of RAPN are influenced by three different types of learning curves including those for the surgical department, the console surgeon, and the assistant. The influence of the bedside assistant clearly has been underestimated to date because it has a significant impact on the perioperative outcomes of RAPN.

**Electronic supplementary material:**

The online version of this article (10.1245/s10434-020-08856-1) contains supplementary material, which is available to authorized users.

Since the first robot-assisted partial nephrectomy (RAPN) in 2005,^1^ this technique has become a standard in high-volume centers with robotic expertise. Nonetheless, RAPN remains a challenging procedure for robotic novices.

For successful implementation and improvement of RAPN programs, different types of learning curves need to be understood. Each surgical department has an overall performance that represents an inherent learning curve. With RAPN, high-volume centers perform better than low-volume centers, but every urologic department should conduct at least 18–20 RAPNs per year to prevent complications.^2^

With a focus on robotic surgeons, Mottrie et al. 3 stated that each robotic surgeon needs to perform more than 30–40 procedures for successful mastery of RAPN. These authors predicted that further improvements should be possible thereafter because learning progress is not complete even after 300 procedures.^4^ The learning curve for RAPN is steeper than for the laparoscopic approach,^5^ but training programs have been developed to improve outcomes.^6^

A third potential learning curve to analyze is that of the bedside assistant who helps to expose the surgical field, applies clips for hemostasis, or assists with dissection. To date, data concerning the impact of the assistant on perioperative outcomes in RAPN have been scarce. Potretzke et al.^7^ compared RAPNs when residents assisted with surgery and did not find any difference. Mitsinikos et al.^8^ showed longer operating times and longer hospital stays but no impact on warm ischemia time or blood loss.

To our knowledge, no study to date has comparatively analyzed the impact of all three learning curves on short-term perioperative outcomes in RAPN. We performed a retrospective, single-center study and included our first 500 consecutive RAPNs to compare the impact of “experience” on perioperative outcomes. For every single operation, experience (EXP) was defined as the current number of RAPNs performed by either our department, the specific surgeon, or the assistant. Patient-related factors also were included in the analysis. Cutoffs to distinguish between “experienced” and “inexperienced” surgical departments, robotic surgeons, and assistants were estimated.

## Patients and Methods

The first 500 consecutive transperitoneal RAPNs in our department from 2007 to 2018 were retrospectively analyzed. The RAPNs were performed using either a DaVinci Si or S system (Intuitive Surgical, Sunnyvale, CA) with a single console. Age, gender, body-mass index (BMI), American Society of Anesthesiology (ASA), and histologic results were obtained as patient-related factors. Tumor complexity according to PADUA^9^ was scored by reviewing preoperative abdominal imaging (computed tomography (CT) or magnetic resonance imaging (MRI)), if available. Operating time, estimated blood loss (EBL), warm ischemia time (WIT), postoperative complications according to Clavien-Dindo grade (within 30 days after surgery), and positive surgical margins (PSMs) served as surgical factors. Conversion rates were divided into conversion rates for either robotic radical, open partial, or open radical nephrectomy. Trifecta rate (absence of PSMs, WIT ≤ 25 minutes, absence of any postoperative complications) and MIC rate (absence of PSMs, WIT ≤ 20 minutes, absence of major postoperative complications ≥ Clavien-Dindo grade 3).^10^,^11^ The presence of “sticky” adherent perinephric fat was scored semiquantitatively from 0 (none) to 2 (massive) by reviewing surgical reports.

The current number of RAPNs performed by either the department, the console surgeon, or the assistant defined EXP. Consequently, each operation had three different EXP values. For example, the 101st RAPN in our department had an EXP of 101 for the department, meaning that the hospital volume for RAPN had reached 101 at this intervention. An EXP of 31 for the console surgeon meant that he or she had reached the 31st RAPN, and an EXP of 1 for the bedside assistant meant he or she had assisted with his or her first case that day.

All the surgeons were consultants assisted by either residents or fellows. All of them had significant prior expertise in performing or assisting with other robot-assisted interventions including pyeloplasties, prostatectomies, and nephrectomies. The surgeons and bedside assistants were paired together upon availability.

As the primary outcome, the study aimed to determine whether the EXP of the department, the surgeon, or the assistant had an impact on the short-term perioperative outcome within 30 days. Furthermore, the effect of tumor- and patient-related factors (PADUA score, presence of sticky fat, BMI, number of prior abdominal surgeries, ASA score, patient age, and sex) on short-term perioperative outcome was assessed. The short-term perioperative outcome was defined by operating time, EBL, WIT, PSM, conversion and complication rates, Trifecta rate, MIC rate, and hospital length of stay.

In the regression analysis, each short-term perioperative outcome parameter served as a dependent variable. The EXP of the department, the surgeon, and the assistant as well as the tumor- and patient-related factors served as independent variables. Independent variables were included in the multiple regression analysis only if the respective effect was significant in the univariate analysis. To compare the influence of the relationships and to analyze whether independent variables were related to each other, Spearman’s rho correlation coefficient rho (*r*) was calculated.

As the secondary outcome, the study aimed to estimate a cutoff value for EXP to differentiate between “experienced” and “inexperienced” surgical departments, console surgeons, and assistants via ROC analysis.

Logistic and linear regression analysis, Fisher’s exact test, the Mann–Whitney *U* test, ROC analysis, and the correlation coefficient according to Spearman‘s rho were calculated using SPSS version 23 (IBM, Armonk, NY, USA). All tests were two-sided, and *p* values lower than 0.05 were considered to indicate significance. This study was approved by the Ethical Review Board of Saarland (reference Bu 67/19, Saarbruecken, Germany). All the study patients provided written consent.

## Results

### Patient Characteristics and Overall Outcome

The patient characteristics and overall surgical outcomes are presented in Table 1. Overall, 7 different surgeons were supported by 37 different assistants. The PADUA score was evenly distributed between low-risk (PADUA 6–7), mid-risk (PADUA 8–9), and high-risk tumors. The RAPN procedure was performed in 157 min, and 82% of the tumors were excised on-clamp within 16 min of WIT. Of the 500 procedures, 40 (8%) were converted, and major postoperative complications according to Clavien-Dindo (grade ≥ 3) occurred in 22 cases (4.4%). Trifecta was achieved in 314 cases (62.8%), and MIC was achieved in 333 cases (66.6%).

**Table 1:** Patient characteristics and the perioperative outcome for 500 consecutive patients from RAPN^a^

Variable	*n* (%)
Age: years (range)	63 (24–93)
Gender	
Male	327 (65.4)
Female	173 (34.6)
BMI: kg/m^2^ (range)	27.6 (18–59.2)
ASA: *n* (range)	2 (1–4)
PADUA: *n* (range)	8 (6–14)
Low-risk	139 (27.8)
Mid-risk	152 (30.4)
High-risk	148 (29.6)
ND	61 (12.2)
Malign histology	363 (72.6)
Clear-cell	265 (73)
Papillary (types 1 and 2)	61 (18.2)
Chromophobe	22 (6)
Other	10 (2.8)
TNM	
pT1	327 (65.4)
pT2	9 (1.8)
≥ pT3	27 (5.4)
Sticky perinephric fat	
0 (none)	354 (70.8)
1 (any)	59 (11.8)
2 (much)	87 (17.4)
Operating time: min (range)	157 (52–376)
EBL: ml (range)	200 (0–2600)
WIT: min (range)	16 (4–43)
Conversion	40 (8)
To open partial nephrectomy	26 (5.2)
To robotic radical nephrectomy	13 (2.6)
To open radical nephrectomy	1 (0.2)
Postoperative complications	122 (24.4)
Minor (Clavien-Dindo 1, 2)	100 (20)
Major (3–5)	22 (4.4)
PSM	32 (6.4)
Trifecta rate	314 (62.8)
MIC rate	333 (66.6)
LOS: days (range)	6 (3–49)

*RAPN* Robot-assisted partial nephrectomy, *BMI* body mass index, *ASA* American Society of Anesthesiology, *ND* not defined, *TNM* tumor-node-metastasis, *EBL* estimated blood loss, *WIT* warm ischemia time, *PSM* positive surgical margin, *LOS* hospital length of stay, *MIC* positive surgical margin, warm ischemia time, postoperative **c**omplications

^a^*n* (%) denotes absolute frequency (%) continuous variables are given as median (range)

### Influence of EXP on Perioperative Outcome

In the multiple regression analysis, EXP of the department showed a strong relation to perioperative outcome parameters. Greater EXP was linked to less WIT, a lower rate of conversion to open partial nephrectomy, and a higher Trifecta rate (all *p* < 0.01). Correlations between EXP and perioperative outcome were weak according to Spearman’s rho (*r* = –0.21; *p* < 0.001; Table 2; Fig. S1).

**Table 2:** Synopsis of impact of experience of the surgical department, the console surgeon, and the bedside assistant on the perioperative outcome in RAPN^a^

	Department		Surgeon		Assistant	
	OR/*B* value	*p* value	OR/*B* value	*p* value	OR/*B* value	*p* value
Operating time: min (range)	–	0.093	− 0.31 (− 0.45 to − 0.17)	< **0.001**	− 0.49 (− 0.93 to − 0.41)	< **0.05**
EBL: ml (range)	–	–	− 1.24 (− 2.05 to − 0.43)	< **0.01**	–	–
WIT time: min (range)	− 0.01 (− 0.02 to − 0.004)	< **0.01**	–	NS	–	–
PSM (%)	–	–	–	–	–	NS
Conversion						
To robotic Nx	–	–	–	–	–	–
To open NSS	0.995 (0.991–0.999)	< **0.05**	–	–	0.94 (0.88 to 0.99)	**<** **0.05**
To open Nx	–	–	–	–	–	–
Absence of						
All complications	–	–	1.01 (1.001 to 1.01)	**0.001**	–	–
Major	–	–	–	0.051	–	–
Trifecta	1.003 (1.002–1.005)	< **0.001**	–	0.13	–	–
MIC	–	0.20	1.01 (1.01 to 1.02)	< **0.001**	1.02 (1.00 to 1.04)	< **0.05**
LOS: days (range)	–	NS	− 0.12 (− 0.02 to 0.002)	< **0.01**	–	NS

*B* value = unstandardized coefficient in regression analysis,

Bold values indicate *p* value < 0.05 were significant

*RAPN* Robot-assisted partial nephrectomy, *OR* odds ratio, *EBL* estimated blood loss, *WIT* warm ischemia time, *NS* not significant, *PSM* positive surgical margin, *Nx* nephrectomy, *LOS* hospital length of stay

^a^The given values were significant in the multiple analysis

The EXP of the console surgeons had a major impact on perioperative outcomes. In the multiple regression analysis, the more experienced surgeons had shorter operating times, less EBL, fewer postoperative complications, higher MIC rates, and shorter hospital stays (all *p* < 0.05). The correlations between EXP and outcome parameters were weak to moderate and strongest between EXP and both operating time (*r* = –0.40; *p* < 0.001) and hospital length of stay (*r* = –0.29; *p* < 0.001; Table 2).

The EXP of the bedside assistants was linked to perioperative outcomes in multiple fashion. The more experienced assistants were associated with shorter operating times, lower conversion rates, and higher MIC rates (all *p* < 0.05). The correlations between EXP and perioperative outcomes were weak to moderate and strongest between EXP and operating time (*r* = –0.23; *p* < 0.001). Fewer PSMs and a shorter hospital stay (both *p* < 0.05) also were linked to greater EXP of the assistants (univariate analysis alone).

The EXPs of the department, the console surgeon, and the assistant correlated with each other in a weak to moderate fashion (0.34 ≤ *r* ≤ 0.6; *p* < 0.001; Table S1). Concerning patient-related factors, only the EXP of the department correlated with sticky fat (*r* = 0.13; *p* < 0.01), and only the EXP of the surgeon correlated with BMI (*r* = –0.12; *p* < 0.01) and patient age (*r* = –0.1; *p* < 0.05). The EXP of the assistant did not correlate with patient-related factors.

### Influence of Patient-Related Factors

The PADUA score was associated with most of the perioperative outcome parameters for all the patient-related factors. Higher PADUA scores were linked to longer WIT (*B* value = 1.68), longer operating times (*B* value = 6.9), greater EBL (*B* value = 39.9), lower Trifecta rate (odds ratio [OR] 0.8), less MIC fulfillment (OR 0.71), and longer hospital stay (*B* value = 0.3) (all *p* < 0.001; Fig. S1).

The adherent perinephric “sticky” fat was associated with more complications (OR 1.28), longer operating times (*B* value = 20.51), and greater EBL (*B* value = 78.5). More obese patients had longer operating times (*B* value = 1.16) (all *p* ≤ 0.05). For further associations, see Fig. S1.

### Cutoff Values for Discrimination Between “Experienced” and “Inexperienced”

**Fig. 1:**
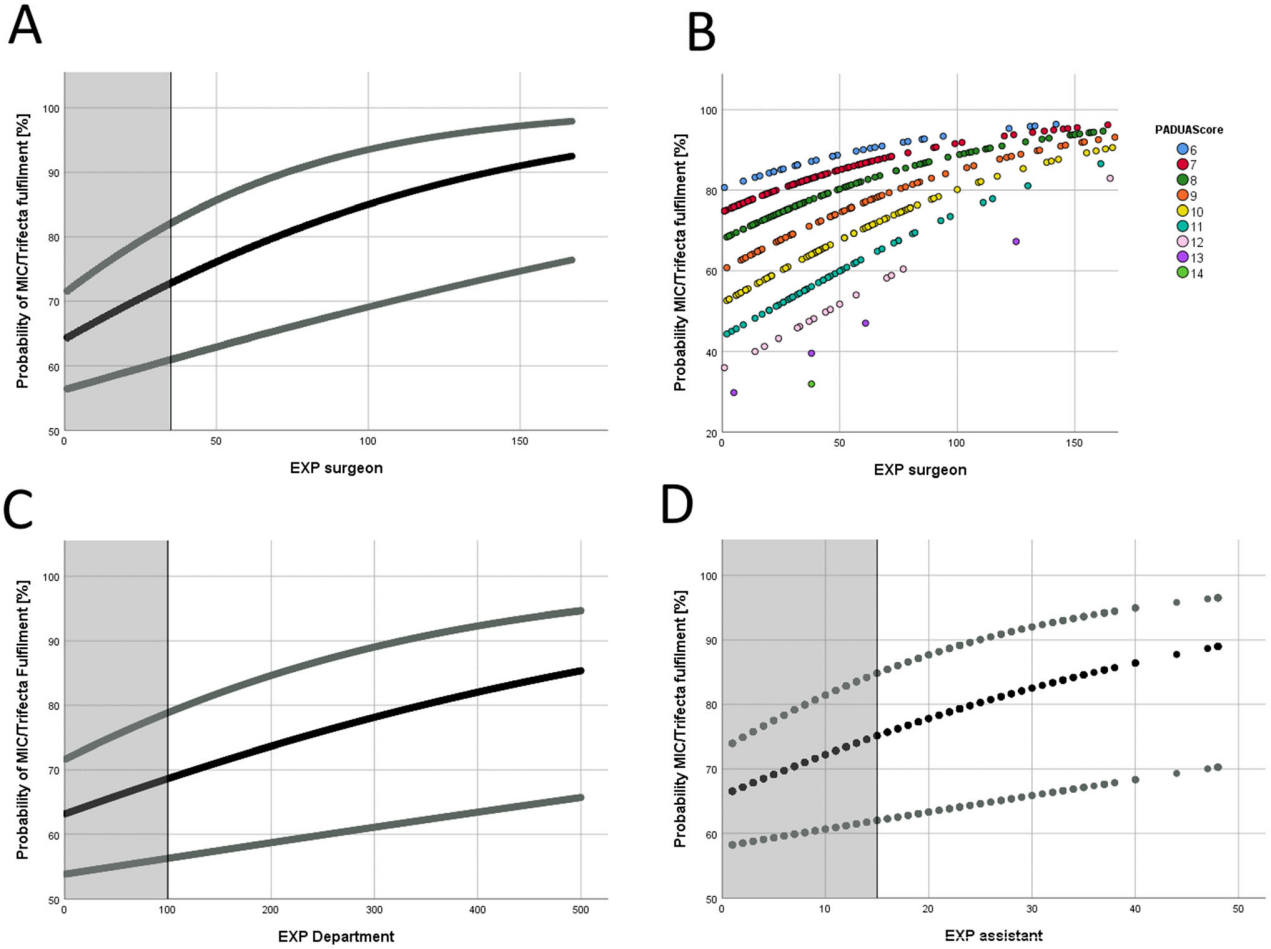
Learning curves of the surgeon, the department, and the bedside assistant. The predicted probability of MIC or Trifecta fulfilment (y-axis) is shown as a function of EXP of either (**a**) the surgeon, (**c**) the department, or (**d**) the bedside assistant (x-axis). The gray lines indicate 95% borders of confidence intervals. The gray area indicates “inexperience.” **b** Learning curve of the surgeon stratified by the PADUA score. MIC, positive surgical margin, warm ischemia time, postoperative complications; EXP, experience

The ROC curves could not precisely define a specific cutoff value for discrimination between “experienced” and “inexperienced.” An EXP greater than 100 for the department, an EXP greater than 35 for the console surgeon, and an EXP greater than 15 for the assistant had the highest Youden indices (Fig. S2). Comparison of the first 100 RAPNs and the next 400 RAPNs in our department showed significantly improved perioperative outcomes and shorter RAPNs with shorter WITs. The rates of conversion to open surgery decreased, and both the Trifecta and MIC rates were achieved significantly more frequently (see Table 3 for detailed data). Therefore, the department was defined as “experienced” with an EXP greater than 100. The console surgeons with EXP greater than 35 performed significantly faster surgeries with less blood loss and shorter WIT during operations on more complex tumors, and were therefore considered “experienced” (Table S2). The RAPN procedure also was shorter when an “experienced” assistant with EXP greater than 15 was present. The result was fewer PSMs, fewer conversions to open partial nephrectomy, and higher Trifecta and MIC rates (Table S2). The individual learning curves for the surgeon, the department, and the bedside assistant indicated a continuous learning process that did not end after 500 procedures (Fig. 1). All cutoffs had in common that the probability to fulfil MIC or Trifecta was approximately 70% for the department, the surgeon, and the assistant when they had become experienced and had reached respectively EXPs of 100, 35, and 15 (Fig. 1).

**Table 3:** Pairwise comparison between the first 100 and next 400 RAPNs for to assess the impact of the department’s experience^a^

Variable	1st 100 RAPNs *n* (%)	Following 400 RAPNs *n* (%)	*p* value
Operating time: min (range)	175 (68–356)	153.5 (52–376)	< **0.001**
EBL: ml (range)	275 (20–2000)	200 (0–2600)	0.08
WIT: ml (range)	19 (0–43)	14 (0–43)	< **0.001**
PSM	5 (0.05)	27 (0.068)	NS
Conversion rate to			
Robotic nephrectomy	1 (1)	12 (3)	NS
Open partial Nx	13 (13)	13 (3.3)	< **0.001**
Open radical Nx	1 (1)	0 (0)	NS
Complication rate			
All complications	31 (31)	91 (22.8)	0.09
Major complications	4 (4)	18 (4.5)	NS
Trifecta rate	49 (49)	265 (66.3)	< **0.01**
MIC rate	49 (49)	284 (71)	< **0.001**
LOS: days (range)	7 (4–26)	6 (3–49)	< **0.01**

Bold values indicate *p* value < 0.05 were significant

*RAPN* Robot-assisted partial nephrectomy, *EBL* estimated blood loss, *WIT* warm ischemia time, *PSM* positive surgical margin, *NS* not significant, *Nx* nephrectomy, *MIC* positive surgical margin, warm ischemia time, postoperative complications, *LOS* hospital length of stay

^a^*n* (%) denotes absolute frequency (%) continuous variables are give a as median (range)

## Discussion

In this study, the first 500 consecutive transperitoneal RAPNs in our department were retrospectively analyzed. The overall performance of our department without prior expertise for any laparascopic partial nephrectomies was comparable with that in the literature. The median operating time was slightly shorter than in two meta-analyses, and the blood loss was similar. The WIT was shorter, and the rate of conversion to open surgery was higher. The complication rates and PSMs were within the range of other studies. Overall, the Trifecta and MIC rates were slightly lower and the hospital stay longer than in other publications.^12^,^13^

These results emerge from different types of learning curves that each exert an independent influence on perioperative outcome. For the first time, the influence of the department, the console surgeon, and the bedside assistant on the perioperative outcome after RAPN was simultaneously assessed. This study defined EXP as the current number of RAPNs performed by each group.

To show an inherent learning curve for the department, the first 100 RAPNs and the next 400 RAPNs were compared. The operating time and WIT decreased, and the conversion rate declined, whereas the Trifecta and MIC rates increased (Table 3). Because the EXP of the department was associated with WIT in the multiple regression analyses, the impact of the department EXP on WIT, the conversion rate, and the Trifecta rate was statistically independent from that of the other factors.

In the multiple regression analysis, the EXP of the console surgeon was associated with shorter operating time, less EBL, fewer complications, higher MIC rate, and shorter hospital stay (Table 2; Fig. S2). Accordingly, Larcher et al.^14^ showed an association of EXP with complication rates and WIT. In contrast, Paulucci et al.^4^ showed no association of EXP with either operating time or complication rates.

The impact of bedside assistants on perioperative results has been rarely assessed to date. According to our analysis, the EXP of the assistant is linked to operating time, risk of conversion, and MIC rate. This relationship was still statistically significant after the multiple analysis. Moreover, it was the only learning curve associated with the PSM rate, albeit only in the univariate regression analysis. Thus, the importance of the assistant in RAPN has clearly been underestimated to date. Some studies describe an association of EXP with complication rates^15^ or longer operating times and hospital length of stay.^8^,^16^ However, the EXP of the assistant correlated with the EXP of the department and the surgeon. This means that the impact of the assistant’s EXP on perioperative outcome was not independent from the EXP of the surgeon or the department (Table S1). In contrast, the EXP of the assistant did not correlate with any patient- or tumor-related factors. Therefore, the impact of the assistant’s EXP on operating time, risk of conversion, and MIC rate as three key outcome parameters in RAPN was independent from patient- and tumor-specific factors, including the PADUA score. Consequently, an inexperienced bedside assistant can cause a longer operating time, a higher risk of conversion, and worse MIC rates in RAPN.

In contrast, the idea of minimizing the impact of bedside assistants has been discussed repeatedly in recent years.^17^ Especially due to the addition of a fourth robotic arm, the fundamental need of an assistant has been questioned.^18^ This concept has been corroborated by multiple studies that did not show an influence of assistants on perioperative outcomes.^7^,^19^ Notably, these studies mainly analyzed robotic radical prostatectomies, a procedure that can be highly standardized. In our opinion, it is feasible to perform a robotic radical prostatectomy without an assistant who handles active parts in surgery because an assistant is needed only for suction or handing of needles. In contrast, RAPN cannot be equally standardized due to the high variability of tumor locations, which render the assistant an irreplaceable part of the team, especially in complex tumor surgery. Thus, performing RAPN without a bedside assistant likely will not be successful. Instead, training programs specifically addressing assistants in RAPN should be developed because comparable programs have proved to be beneficial in robot-assisted radical prostatectomies.^20^,^21^

With regard to the analyses of Vickers et al.^22^,^23^ focused on learning curves in radical prostatectomies, we calculated three distinct learning curves for the console surgeon, the department, and the bedside assistant (Fig. 1). These indicate the probability of MIC or Trifecta fulfilment as a function of EXP and illustrate that the learning process in RAPN is continuous and does not end even after 500 procedures. For this reason, it is not possible to calculate a definite cutoff for EXP using the Youden index because neither the department, the surgeon, nor the assistant stop learning. Nonetheless, our cutoffs for EXP (department EXP > 100, surgeon EXP > 35, assistant EXP > 15) are highly robust, with the department, the surgeon, and the assistant performing significantly better when they have become “experienced” (Tables 3 and S2). Furthermore, all cutoffs have in common that the probability of either the department, the surgeon, or the assistant fulfilling MIC or Trifecta is about 70%, when they have become “experienced” (Fig. 1).

Our analysis also showed that several patient-related factors exert an important influence on perioperative outcomes in RAPN. Apart from the RENAL score and the c-index, the PADUA score is one of the most common renal tumor complexity scores.^9^,^24^,^25^ According to other works, the PADUA score correlates with multiple peri- and postoperative outcome parameters in RAPN.^26^,^27^

In our study, the PADUA score showed a significant association with operating time, EBL, WIT, Trifecta rate, MIC rate, and hospital length of stay in the multiple analysis. Therefore, the influence of tumor complexity on perioperative outcomes is comparable with that of console surgeons. Stratification of the console surgeon’s learning curve by the PADUA score showed a tremendous impact (Fig. 1b). The probability of MIC or Trifecta fulfilment for a surgeon with an EXP of 50 was approximately 90% when operating on a PADUA 6 tumor, but only 50% when operating on a PADUA 12 tumor and therewith lowered by 40%.

In this study, 90 potential relationships were included for a comparative assessment of the impact that individual learning curves and patient-related factors have on perioperative outcomes in RAPN. Almost 30 associations were significant in the multiple analysis (Fig. S1). No learning curve and no patient-related factors showed an impact on all perioperative outcome parameters simultaneously. Regardless, the EXP of the surgeon and the PADUA score have an impact on most parameters and can therefore be considered as the factors with the most important influence on perioperative outcomes in RAPN. Nonetheless, the coefficients showing the highest correlation with the perioperative outcome parameters were comparably low. This finding highlights the importance of all other influencing factors in parallel, mainly the EXP levels of the department and the assistant.

This monocentric and retrospective study was not devoid of limitations. The main focus of this work was on showing the impact of EXP in one distinct procedure on perioperative outcome, with EXP defined as the current number of RAPNs performed by either the department, the console surgeon, or the assistant. In contrast, each resident’s individual year of training representing his or her total surgical capabilities was not considered. In this real-world analysis, case mix changed over time as the PADUA score slightly increased. Therefore, regression analysis was stratified for the PADUA score. Not least, it might have been possible to analyze even more potential influencing factors, but this would have made the interpretation of our results even more difficult.

In summary, RAPN remains a challenging procedure despite its wide acceptance. One main reason is the simultaneous influence of learning curves and patient-related factors on perioperative outcomes, which are nearly impossible to control all at once. The EXP not only of the console surgeon but also of the whole department and the assistant influence RAPN outcomes. The influence of the bedside assistant clearly has been underestimated to date. As a consequence, training strategies for the department, the console surgeons, and the assistants as well as patient selection are key to fast and sustainable success in RAPN. Based on these results, we desire improvement of our training structure for bedside assistants to overcome potential detrimental effects.

## Electronic supplementary material

Below is the link to the electronic supplementary material.

Supplementary material 1 (DOCX 945 kb)
