# Supplementary material for: Three Different Learning Curves Have an Independent Impact on Perioperative Outcomes After Robotic Partial Nephrectomy: A Comparative Analysis
Source: Ann Surg Oncol. 2020 Jul 24;28(2):1254–61. doi: 10.1245/s10434-020-08856-1 (PMC7801306; doi:10.1245/s10434-020-08856-1)

**Table S1:** Correlation analysis between EXP of the department, surgeon and assistant with patient-related factors.

|  | **EXP department** | **EXP**  **surgeon** | **EXP**  **assistant** | PADUA | sticky fat | BMI | prior surgery | ASA | patient age | sex |
| --- | --- | --- | --- | --- | --- | --- | --- | --- | --- | --- |
| **EXP department** |  | r=0.598  p<0.001 | r=0.5  p<0.001 | n.s. | r=0.131  p<0.01 | n.s. | n.s. | n.s. | n.s. | n.s. |
| **EXP**  **surgeon** | r=0.598  p<0.001 |  | r=0.336  p<0.001 | n.s. | n.s. | r=-0.117  p<0.01 | n.s. | n.s. | r=-0.091  p<0.05 | n.s. |
| **EXP**  **assistant** | r=0.5  p<0.001 | r=0.336  p<0.001 |  | n.s. | n.s. | n.s. | n.s. | n.s. | n.s. | n.s. |

**Table S2**: Comparison of the perioperative results of experienced vs. inexperienced console surgeons (> 35 surgeries) and bed-side assistants (> 15 surgeries). Median (range) are given as well as absolute frequency (percentage).

|  | **console surgeon** | | | **bed-side assistant** | | |
| --- | --- | --- | --- | --- | --- | --- |
|  | inexperienced  (n=215) | experienced  (n=285) | p-value | inexperienced  (n=283) | experienced  (n=217) | p-value |
| operating time [min] | 175 (52-376) | 141 (57-327) | **<0.001** | 165 (57-376) | 138.5 | **<0.001** |
| EBL [ml] | 250 (0-1800) | 200 (0-2600) | **<0.001** | 235 (0-2000) | 200 (0-2600) | n.s. |
| WIT [min] | 16 (0-43) | 14 (0-43) | **<0.01** | 15 (0-43) | 14 (0-40) | n.s. |
| PSM | 18 (8.4%) | 14 (4.9%) | n.s. | 26 (0.01%) | 6 (0.03%) | **<0.01** |
| conversion to |  |  |  |  |  |  |
| robotic nephrectomy | 6 (2.8%) | 7 (2.5%) | n.s. | 5 (1.8%) | 8 (3.7%) | n.s. |
| open partial Nx | 16 (7.4%) | 10 (3.5%) | n.s. | 23 (8.1%) | 3 (1.4%) | **<0.001** |
| open radical Nx | 0 | 1 (0.4%) | n.s. | 1 (0.4%) | 0 | n.s. |
| complication rate |  |  |  |  |  |  |
| all complications | 64 (29.8%) | 58 (20.4%) | **<0.05** | 72 (25.4%) | 50 (23%) | n.s. |
| major complications | 14 (6.5%) | 9 (3.2%) | n.s. | 14 (4.9%) | 8 (3.7%) | n.s. |
| Trifecta rate | 115 (53.5%) | 199 (69.8%) | **<0.001** | 166 (58.7%) | 148 (68.2%) | **<0.05** |
| MIC rate | 125 (58.1%) | 208 (73%) | **<0.001** | 171 (60.4%) | 162 (74.7%) | **<0.001** |
| length of stay [d] | 7 (4-49) | 6 (3-29) | **<0.001** | 6 (3-49) | 6 (4-29) | n.s. |

**Figure S1:** Synopsis of the relationship between experience and patient-related factors with perioperative outcome parameters. Here, only relations that are significant in multiple regression analysis are shown. Color code indicates the strength of the correlation according to Spearman‘s Rho coefficient, scaled from light gray (lowest correlation strength according to Spearman’s Rho) to black (highest).

|  | | **perioperative outcome** | | | | | | | | |
| --- | --- | --- | --- | --- | --- | --- | --- | --- | --- | --- |
|  |  | operating time | estimated blood loss | warm ischemia time | positive surgical margin | conversion | complications | Trifecta | MIC | length of stay |
| **experience EXP** | **department** |  |  | r=-0.21  p<0.001 |  | p<0.001 |  | p<0.001 |  |  |
|  | **surgeon** | r= -0.40  p<0.001 | r= -0.22  p<0.001 |  |  |  | p<0.01 |  | p<0.001 | r= -0.29  p<0.001 |
|  | **assistant** | r= -0.23  p<0.001 |  |  |  | p<0.001 |  |  | p<0.01 |  |
|  |  |  |  |  |  |  |  |  |  |  |
| **patient-related factor** | PADUA | r=0.15  p<0.01 | r=0.17 p<0.001 | r=0.32 p<0.001 |  |  |  | p<0.01 | p<0.001 | n.s  p=0.054 |
|  | sticky fat | r=0.23  p<0.001 | r=0.16  p<0.001 |  |  |  | p<0.05 |  |  |  |
|  | BMI | r= 0.21  p<0.001 |  |  |  |  |  |  |  |  |
|  | prior surgery |  |  |  |  |  |  |  |  |  |
|  | ASA |  | r=0.17  p<0.001 |  |  |  |  | p<0.01 |  | r=0.17  p<0.001 |
|  | patient age |  |  |  |  | p<0.05 |  |  |  | r=0.21  p<0.001 |
|  | sex | n.s.  p=0.054 |  |  |  |  |  |  |  | r=0.12  p<0.001 |

**Figure S2:** Representative ROC curve to define a cut-off value to differentiate “experienced” from “inexperienced” surgeon by Trifecta fulfilment. The relationship between EXP of the surgeon and Trifecta fulfilment is analyzed.


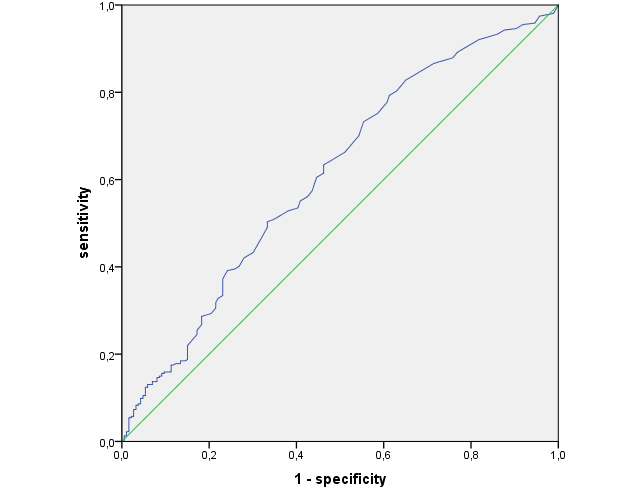

Supplement: Supplementary file 1 — Supplementary material 1 (DOCX 945 kb) [file 10434_2020_8856_MOESM1_ESM.docx]
